# Comparison of therapeutic evaluation criteria in FDG-PET/CT in patients with diffuse large-cell B-cell lymphoma: Prognostic impact of tumor/liver ratio

**DOI:** 10.1371/journal.pone.0211649

**Published:** 2019-02-07

**Authors:** Mathieu N. Toledano, Pierre Vera, Hervé Tilly, Fabrice Jardin, Stéphanie Becker

**Affiliations:** 1 Nuclear Medicine Department, Henri Becquerel Cancer Center, Rouen, France; 2 QuantIF–LITIS (EA 4108-FR CNRS 3638), Faculty of Medicine, University of Rouen, Rouen, France; 3 INSERM U1245, Centre Henri Becquerel, Rouen, France; 4 Hematology department, Centre Henri Becquerel, Rouen, France; Ente Ospedaliero Cantonale, SWITZERLAND

## Abstract

**Purpose:**

The study objective was to compare the prognostic value of interim and end-of-treatment FDG PET/CT using five therapeutic evaluation criteria in patients with diffuse large B cell lymphoma (DLBCL).

**Methods:**

181 patients were retrospectively analysed. All patients underwent FDG-PET at baseline and after four cycles (iPET4) of first-line chemotherapy and 165 at the end-of-treatment (PET-eot). Ratio Deauville score (rDS) (SUVmax-target residual lesion/SUVmax-liver) was measured in iPET4 and PET-eot, and its optimal threshold was determined using receiver operating characteristic (ROC) curve analysis. Deauville score (DS) (iPET4 and PET-eot), ΔSUVmax, ΔSUVmax determined according to Menton 2011 criteria (ΔSUVmax+DS) and ΔSUVmax+rDS were also evaluated (iPET4 only). Median follow-up was 44 months.

**Results:**

ROC analysis revealed the optimal cut-off value was 1.4-fold of SUVmax-liver on iPET4 and PET-eot. On iPET4, positive predictive value (PPV) of rDS was significantly better than DS: 81.58% vs. 67.79%. In univariate analysis, the five interpretation methods were statistically significant (p<0.0001 for progression-free survival [PFS] and overall survival [OS]). In multivariate analysis, only rDS was an independent prognostic factor (p = 0.0002 and p<0.0001 for PFS and OS, respectively). On PET-eot, similarly, the two therapeutic evaluation criteria analysed (rDS and DS) were statistically significant at the univariate level (p<0.0001). rDS was the only significant prognostic factor in multivariate analysis (p<0.0001). PPV and accuracy of rDS were also better than DS.

**Conclusions:**

rDS with a tumor/liver ratio of 1.4 is a robust prognostic factor in patients with DLBCL on iPET4 and PET-eot.

## Introduction

Despite the improvement made by immunochemotherapy, 30% to 40% of patients diagnosed with large-B-cell lymphoma (DLBCL) will relapse [1], with a majority of patients dying of the disease [2]. It is therefore crucial to identify these nonresponder patients to offer them new treatments. Fluorodeoxyglucose positron emission tomography/computed tomography (FDG-PET/CT) has been widely validated as a prognostic tool in DLBCL and Hodgkin lymphoma (HL) for both interim and end-of-treatment [3–7]. Interpretation criteria have evolved to decrease false positives, and the current recommendations are to use Deauville criteria (5-point scale-Deauville Score [DS]) with a positive PET defined by tumor residual uptake moderately higher than liver (score 4) or two to three times the liver SUVmax (score 5). These criteria are applicable for both interim and end-of-treatment PET [3]. However, at present, changing treatment solely based on interim PET (iPET) is not recommended outside clinical trials [3]. Indeed, a negative iPET is truly negative (e.g., high negative predictive value [NPV]) in most cases, whereas positive iPET may be truly or falsely positive, with a high percentage of patients with abnormal uptake presenting a good outcome [8]. Studies using Deauville criteria analysis with a cut-off >3 for iPET in DLBCL reported good NPV with 2-year PFS rates between 84% and 85%, whereas the PPV ranges from 25% to 55% [9–11]. To improve iPET’s PPV, a quantitative approach was proposed after 2 and 4 courses of chemotherapy with a ΔSUVmax based on the reduction of tumor SUVmax from baseline. The ΔSUVmax method has shown to be more reproducible between readers and more robust than Deauville criteria by decreasing the number of false positives [4–7]. The PETAL study, including 600 patients with DLBCL, demonstrated that the ΔSUVmax approach with a cut-off of 66% of reduction (iPET2) was highly predictive of outcome [12]. However, it does not appear effective for low-risk (International Prognostic Index [IPI] ≤1) patients, for whom the DS is accurate [13]. In addition, the determination of ΔSUVmax has been modified according to Menton 2011 criteria [14], and these modified criteria were never validated (ΔSUVmax if eligible patient and DS if SUVmax initial tumor <10 and or if SUVmax residual tumor >5). Several authors suggested a quantitative adaptation of the DS to better separate a score 3 and 4 in HL [15, 16], DLBCL [17–19] and follicular lymphoma (FL) [20], using a ratio between the residual uptake and the liver. The aim of this study was to compare the prognostic value of these different therapeutic evaluation criteria after four courses of immunochemotherapy (iPET4) and at the end-of-treatment PET (PET-eot) in patients with DLBCL: ratio Deauville score (rDS), visual DS, ΔSUVmax, ΔSUVmax determined according to Menton 2011 criteria and ΔSUVmax + rDs.

## Methods

### Study population

This monocentric study was approved by the Henri Becquerel centre review board (n°1804B). Patients were informed about the use of anonymized data for research and their right to oppose this use. The study enrolled 181 patients between July 2005 and September 2014, who were retrospectively evaluated. The inclusion criteria were as follows: DLBCL confirmed in all patients by a histopathologic review of a baseline biopsy, treatment using an anthracycline-containing regimen with rituximab; R-CHOP chemotherapy (Rituximab, cyclophosphamide, doxorubicin, vincristine, prednisolone) or R-CHOP-like, including R-miniCHOP, R-COPADEM (methotrexate, cyclophosphamide, vincristine, doxorubicin, prednisolone) and R-ACVBP (doxorubicin, vindesine, cyclophosphamide, bleomycin, prednisolone), staging with FDG-PET/CT at baseline (PET0) and after four cycles (iPET 4) of chemotherapy and a follow-up by at least 2 years after completion of first-line therapy. Clinical data obtained from all patients included the following: sex, age at disease onset, Eastern Cooperative Oncology Group (ECOG) performance status, extranodal disease, Ann Arbor staging system and lactate dehydrogenase (LDH) level; this allowed us to calculate IPI score.

### FDG-PET/CT acquisitions

PET0, iPET4 and PET-eot were performed after a 6-hour fast, and blood glucose level was less than 1.7 g/l before injection of the radiotracer.

4.5 MBq/kg of FDG were injected after 30 min of rest. Sixty minutes later (±5 min), acquisitions began with a CT scan in the craniocaudal direction. CT scan parameters were set to 120 kV and 100–150 mAs (based on patient’s weight) using the dose reduction software (CareDose, Siemens Medical Solutions, Hoffman Estates, Knoxville, TN, USA). This yielded a mean effective mA s of 89.1 ± 6.7. The patient’s arms were positioned over their head, and acquisition was performed with free breathing and a 16 x 0.75-mm primary collimation. The duration of the CT scan was 20 s. No contrast media injection was done. PET image acquisitions immediately followed in the caudocranial direction, and the scan time was based on 3 min per bed position. Six to eight positions were acquired (whole body); the axial field of view for 1 bed position was 162 mm with a bed overlap of 25% (plane spacing: 2 mm). The transverse spatial resolution reached 4.4 mm (centred point source in air).

Baseline, interim and end-of-treatment scans were performed on the same PET/CT. All scans were displayed using a fixed standardized uptake value (SUV) scale and colour table. Two nuclear medicine physicians (MNT and SB), who were blinded to clinical outcome, reviewed the paired scans.

### FDG-PET/CT measurement and interpretation

On PET0 images, the tumor with the most intense uptake was carefully identified and measured using a fixed SUV scale and colour table. On iPET4 images, if residual tumor was present, SUVmax was measured on the most intense focus even though its location differed from the most intense tumor on the PET0 image. In images in which no lesions were identifiable, SUVmax was considered equal to 1 (i. e. the value in case of homogeneous distribution of FDG over the whole organism). The percentage decrease in SUVmax between PET0 and iPET4 (ΔSUVmax) was calculated, and a cut-off value of 70% was used to separate good from bad responders [6]. The iPET4 and PET-eot scans were visually interpreted using the Deauville five-point scale compared with the PET0 scans: score 1, no residual uptake; score 2, uptake ≤ mediastinum; score 3, uptake > mediastinum but ≤ liver; score 4, uptake moderately > liver; score 5, uptake> two to three times the liver SUVmax and/or progression of the lesions [3]. Liver measurement was assessed by placing a spherical volume of interest (VOI) of diameter 3 cm in the right upper lobe of the liver, avoiding the edge and any single ‘hot’ pixels likely to represent noise, sampling several axial slices to obtain a representative maximum liver SUVmax [21]. VOI of the mediastinum blood pool was drawn just above the aortic root, avoiding the vessel wall and any areas of calcification. rDS was defined as the ratio between SUVmax of the hottest target residual lesion and SUVmax of the liver right lobe and was measured on iPET4 and PET-eot. Furthermore, two others interpretation methods had been tested. ΔSUVmax determined according to Menton 2011 criteria (ΔSUVmax if eligible patient and DS if SUVmax initial tumor <10 and/or if SUVmax residual tumor >5 –ΔSUVmax + DS) and ΔSUVmax + rDS (ΔSUVmax if eligible patient and rDS if SUVmax initial tumor <10 and/or if SUVmax tumor residual >5).

### Statistical analysis

Statistical analysis was conducted using MedCalc (MedCalc Software). OS and PFS were defined according to the revised National Cancer Institute (NCI) criteria [22]. Interobserver agreement was evaluated by Cohen’s kappa coefficient. Univariate cox regression analysis was performed to determine which measures were predictive of PFS and OS. Variables which were significantly associated with PFS and OS in univariate analysis were included in multivariate analysis to identify measures independently predictive of survival. The ROC analysis was applied on continuous variables to identify the optimal cut-off values. Only DS 3 and 4 were considered to determine the optimal cut-off. Then the cut-off was evaluated in the whole population. Time-to-event endpoints were evaluated by Kaplan–Meier survival curves and compared using the Log rank test. Statistical significance was considered at p <0.05 and was controlled by Altman (continuous variables only) and Benjamini-Hochberg procedures.

## Results

### Patient characteristics

We retrospectively enrolled 181 patients with DLBCL. Median follow-up was 44 months (range 26–71 months). The clinical characteristics are summarized in **Table 1**. Seventy patients progressed or relapsed, and 55 patients died, with a median delay of 11 and 18 months, respectively. The 5-year PFS and OS rates for the whole group were 61% and 68%, respectively. All patients were treated by R-CHOP or R-CHOP-‘like’ regimens.

**Table 1:** Patient clinical characteristics in the whole population.

Patient Characteristics		n = 181 (100%)
Gender	Female	92 (51)
	Male	89 (49)
Age at diagnosis	Median (range)	62 (18–87) years
Follow-up	Median	44 months
Chemotherapy	R-CHOP	96 (53)
	R-miniCHOP	20 (11)
	R-COPADEM	3 (2)
	R-ACVBP	62 (34)
ECOG performance status	0	91 (50)
	1	52 (29)
	2	20 (11)
	3	15 (8)
	4	3 (2)
Ann Arbor stage	I-II	40 (22)
	III-IV	141 (78)
LDH	Normal	56 (31)
	Elevated (>480)	125 (69)
IPI score	Low (0–1)	39 (22)
	Low-intermediate (2)	50 (28)
	High-intermediate (3)	55 (30)
	High (4–5)	37 (20)

### ROC analysis

**Fig 1:**
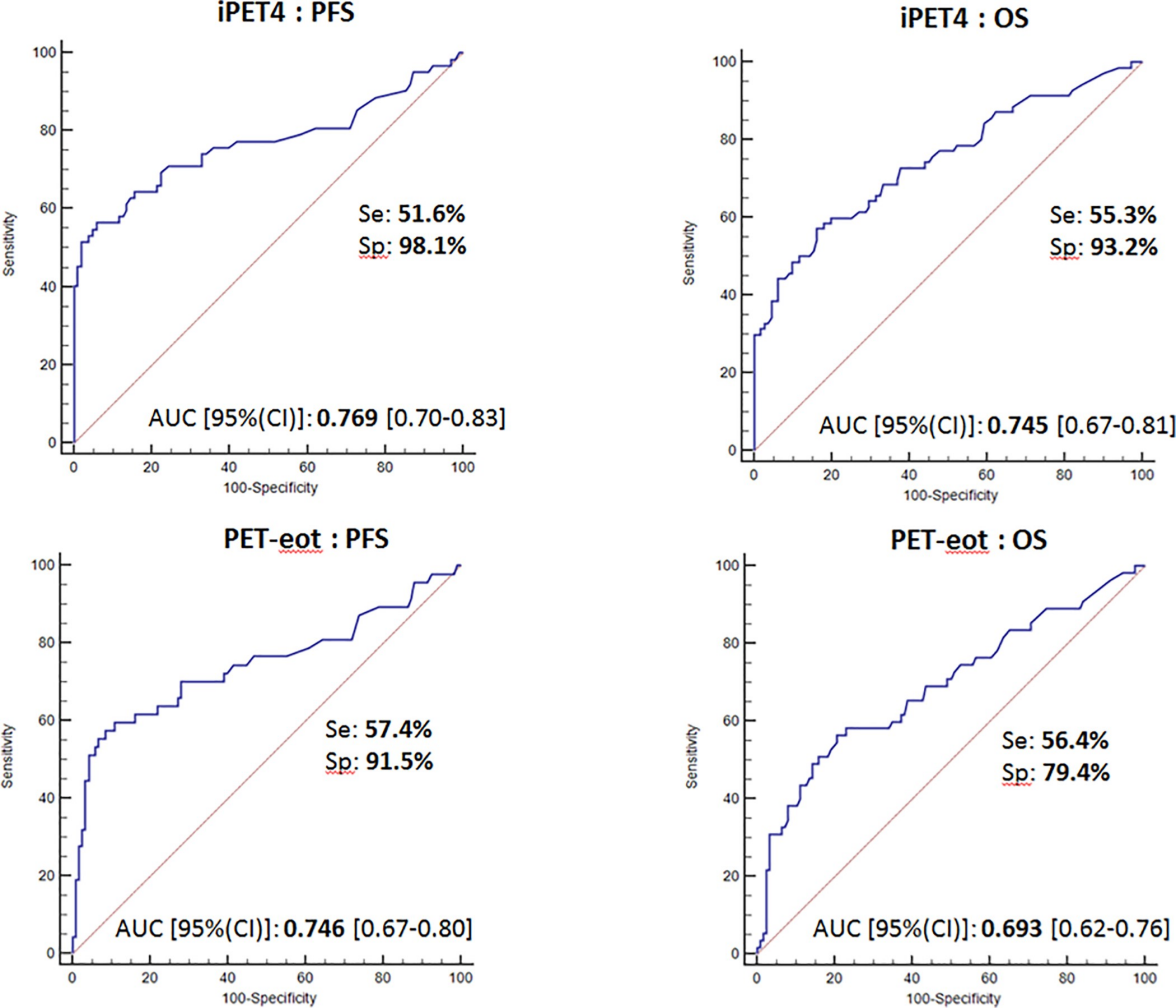
ROC analysis of the Tumor/Liver SUVmax ratio (rDS).

ROC analysis (**Fig 1**) showed that the optimal cut-off for rDS was 1.38-fold of SUVmax-liver on iPET4 and 1.36- fold of SUVmax liver on PET-eot (sensitivity 51.6% and 57.4%, specificity 98.1% and 91.5% for PFS, sensitivity 55.3% and 56.4%, specificity 93.2% and 79.4% for OS). Areas under the curve (AUC) showed a robust prognostic accuracy (0.769 and 0.746 with p<0.0001 for PFS and 0.745 and 0.693 with p<0.0001 for OS, on iPET4 and PET-eot respectively).

There were no outcome changes between 1.38 and 1.4 for iPET4 and between 1.36 and 1.4 for PET-eot. For simplicity, we have rounded these values to 1.4 for iPET4 and PET-eot.

### Interobserver agreement

#### iPET4

Interobserver agreement was near perfect, with SUVmax-liver- based interpretation (к = 0.93) and DS (к = 0.85) and perfect for ΔSUVmax (к = 1) (**Table 2**).

**Table 2:** Interobserver agreements for interim and end-of-treatment PET/CT with use of rDS, Deauville score and ΔSUVmax.

	Observer 1 vs. 2	
	iPET4	PET-eot
**Tumour/Liver ratio (rDS)**	0.93	0.84
**Deauville score**	0.85	0.56
**ΔSUVmax**	1.00	-

Cohen’s kappa coefficient κ interpretation: 0.00–0.20, slight agreement; 0.21–0.41, fair agreement; 0.41–0.60, moderate agreement; 0.61–0.80, substantial agreement; 0.81–1.00, almost perfect agreement. rDS: ratio Deauville Score = Tumour/Liver SUVmax ratio >1.4 as positive for iPET4 and PET-eot; Deauville score: score ≥ 4 as positive for iPET4 and PET-eot; ΔSUVmax: ≤70% as positive for iPET4.

#### PET-eot

Interobserver agreement was also near perfect with rDS (к = 0.84), but a moderate agreement was found for DS (к = 0.56) (**Table 2**).

### Prognosis accuracy of different therapeutic evaluation criteria

#### iPET4

Fifty-two patients (29%) were not eligible for evaluation by ΔSUVmax according to Menton 2011 criteria (SUVmax initial tumor <10 and/or SUVmax residual tumor >5).

rDS, Menton 2011 criteria and ΔSUVmax had the best accuracy, which was better than DS and ΔSUVmax+rDS. They could be applied in our population with a better specificity but a relatively lower sensitivity compared to DS. ΔSUVmax + rDS had the same specificity as Menton 2011 criteria. NPVs were high for all evaluation criteria on PFS and OS for iPET4. rDS, Menton 2011 criteria and ΔSUVmax had robust PPVs for PFS but moderate for OS, even if better than DS. The use of rDS has allowed a significantly increased PPV compared to Deauville criteria and therefore has decreased the number of false positives (7 vs. 19 patients for PFS and 14 vs. 28 patients for OS) (**Table 3**).

**Table 3:** Prognosis accuracy of interim and end-of-treatment PET/CT interpreted using different therapeutic evaluation criteria.

	Sensitivity	Specificity	PPV	NPV	Accuracy
**iPET4 (n = 181)**					
PFS					
**rDS**	44.28%	93.69%	81.58%	72.72%	74.58%
**ΔSUVmax**	41.43%	94.59%	82.85%	71.92%	74.03%
**Deauville Score**	57.14%	82.88%	67.79%	75.41%	72.93%
**ΔSUVmax + rDS**	41.43%	92.79%	78.38%	71.53%	72.93%
**Menton 2011 criteria**	45.71%	92.79%	80.00%	73.05%	74.58%
OS					
**rDS**	43.63%	88.89%	63.16%	78.32%	75.14%
**ΔSUVmax**	40.00%	89.68%	62.85%	77.39%	74.58%
**Deauville Score**	56.36%	77.78%	52.54%	80.32%	71.27%
**ΔSUVmax + rDS**	40.00%	88.09%	59.46%	77.08%	73.48%
**Menton 2011 criteria**	45.45%	88.09%	62.50%	78.72%	75.14%
**PET-eot (n = 165)**					
PFS					
**rDS**	51.61%	98.06%	94.12%	77.10%	80.60%
**Deauville Score**	58.06%	88.35%	75.00%	77.77%	76.96%
OS					
**rDS**	55.32%	93.22%	76.47%	83.97%	82.42%
**Deauville Score**	61.70%	83.90%	60.42%	84.61%	77.57%

PPV: positive predictive value; NPV: negative predictive value; PFS: progression-free survival

OS: overall survival.

rDS: ratio Deauville Score = Tumour/Liver SUVmax ratio >1.4 as positive for iPET4 and PET-eot

Deauville Score: score≥ 4 as positive for iPET4 and PET-eot; ΔSUVmax: ≤ 70% as positive for iPET4

ΔSUVmax + rDS: ΔSUVmax if eligible patient and rDS if SUVmax initial tumour <10 and / or if SUVmax residual tumour >5.

Menton 2011 criteria: ΔSUVmax if eligible patient and DS if SUVmax initial tumour <10 and / or if SUVmax residual tumour >5.

**Fig 2:**
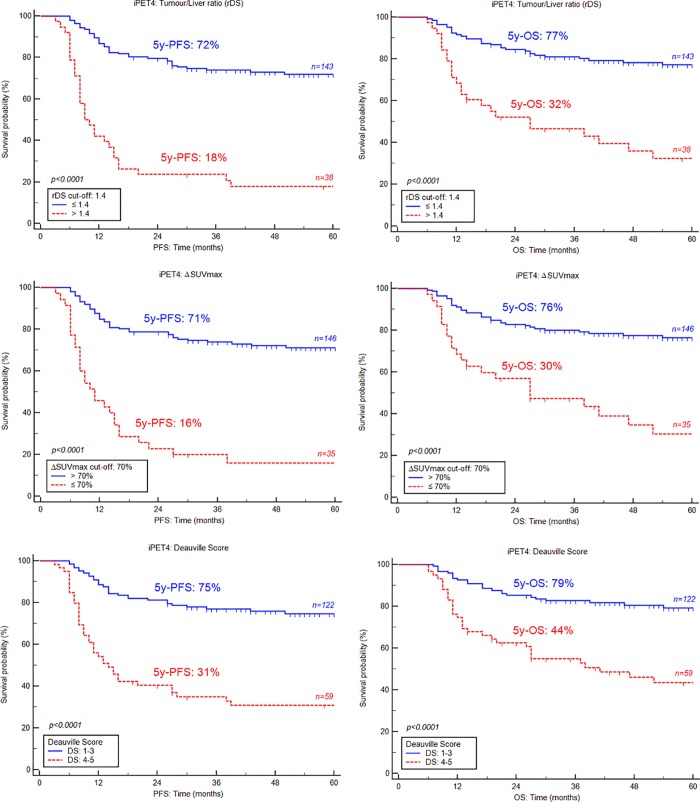
Kaplan-Meier estimates of progression-free and overall survival (PFS, OS) on iPET4 according to the Tumor/Liver ratio (rDS), ΔSUVmax and Deauville score.

Kaplan–Meier curves show that iPET4, using any of therapeutic evaluation criteria, was a strong prognostic factor for both PFS and OS (p<0.0001) (**Fig 2**). When the rDS was used, the 5-year PFS and OS were 18% and 32%, respectively, in iPET4-positive patients vs. 72% and 77%, respectively, in iPET4-negative patients (p<0.0001).

#### PET-eot

rDS and DS were analysed. rDS had better accuracy than DS on PFS and on OS. rDS had a better specificity and therefore a better PPV. At the end-of-treatment evaluation, again the number of false positives decreased significantly for the benefit of rDS compared to DS (2 vs. 12 patients for PFS and 8 vs. 19 patients for OS). Inversely, DS had a better sensitivity, but NPV was similar for both PFS and OS **(Table 3).**

**Fig 3:**
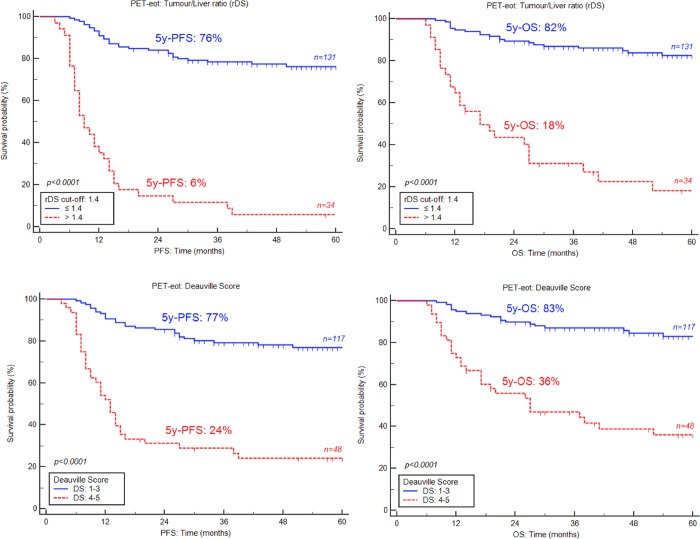
Kaplan-Meier estimates of progression-free and overall survival (PFS, OS) on PET-eot according to the Tumor/Liver ratio (rDS) and Deauville score.

A statistically significant difference on PFS and OS was observed between patients whose rDS was higher and lower than the 1.4 cut-off. The 5-year PFS for patients with negative PET was 76% vs. 6% for positive PET, and 5-year OS were 82% vs. 18% with p<0.0001 for both PFS and OS. Survival rates using DS were statistically significant but less than rDS for nonresponder patients. Indeed, 5-year PFS and OS were 77% vs. 24% and 83% vs. 36% respectively (p< 0.0001 for both PFS and OS)(**Fig 3**).

### Univariate and multivariate analysis

#### Univariate analysis

**iPET4:** Univariate cox regression analysis showed that the following parameters were statistically significant for PFS and OS: rDS, DS, ΔSUVmax, Menton 2011 criteria, ΔSUVmax + rDS, IPI score, chemotherapy and LDH rate. ECOG Performance Status (PS) was significant only for OS, and Ann Arbor stage was not significant at univariate level **(Table 4).**

**Table 4:** Univariate analysis and log-rank test controlled by Benjamini-Hochberg correction for PFS and OS at interim and end-of-treatment PET/CT.

	Univariate analysis					
iPET4 (n = 181)	PFS			OS		
Variables	Cox regression analysis		Log rank test	Cox regression analysis		Log rank test
	HR	95%CI	p-value*	HR	95%CI	p-value*
**rDS**	5.57	[3.45–8.99]	<0.0001	4.08	[2.39–6.95]	<0.0001
**Deauville score**	4.21	[2.61–6.76]	<0.0001	3.47	[2.04–5.92]	<0.0001
**ΔSUVmax**	5.39	[3.33–8.75]	<0.0001	3.87	[2.25–6.65]	<0.0001
**ΔSUVmax + rDS**	4.82	[2.98–7.81]	<0.0001	3.54	[2.08–6.09]	<0.0001
**Menton 2011 criteria**	5.19	[3.22–8.38]	<0.0001	4.13	[2.42–7.05]	<0.0001
**IPI score**	3.36	[1.98–5.68]	<0.0001	3.74	[2.01–6.94]	<0.0001
**Chemotherapy**	3.55	[1.87–6.74]	<0.0001	4.34	[1.97–9.56]	0.0001
**LDH**	2.05	[1.14–3.68]	0.013	2.84	[1.34–5.99]	0.004
**ECOG PS**	1.57	[0.92–2.68]	0.092	1.88	[1.05–3.36]	0.029
**Ann Arbor**	1.68	[0.88–3.20]	0.1	1.81	[0.86–3.82]	0.11
**PET-eot (n = 165)**						
**rDS**	10.31	[6.12–17.36]	<0.0001	8.61	[4.79–15.48]	<0.0001
**Deauville score**	5.92	[3.55–9.89]	<0.0001	5.71	[3.16–10.32]	<0.0001
**IPI score**	3.39	[1.94–5.94]	<0.0001	3.86	[1.96–7.59]	0.0001
**Chemotherapy**	3.12	[1.62–5.99]	0.0006	3.64	[1.63–8.13]	0.0016
**LDH**	2.12	[1.15–3.90]	0.016	3.02	[1.35–6.74]	0.007
**ECOG PS**	1.48	[0.81–2.69]	0.19	1.77	[0.92–3.41]	0.088
**Ann Arbor**	1.43	[0.74–2.75]	0.28	1.48	[0.69–3.17]	0.31

* Statistical significance was considered at p < 0.05 and was controlled by Benjamini-Hochberg procedure.

PFS: Progression Free survival; OS: Overall Survival; rDS: ratio Deauville Score = Tumour/Liver SUVmax ratio

Menton 2011 criteria: ΔSUVmax if eligible patient and DS if SUVmax initial tumour <10 and / or if SUVmax residual tumour >5

ΔSUVmax + rDS: ΔSUVmax if eligible patient and rDS if SUVmax initial tumour <10 and / or if SUVmax residual tumour >5

IPI: International Prognostic Index; LDH: Lactate Dehydrogenase; ECOG PS: Eastern Cooperative Oncology Group Performance Status.

**PET-eot:** In the univariate analysis, rDS, DS, IPI score, chemotherapy and LDH were statistically significant for PFS and OS. ECOG PS and Ann Arbor stage were not associated with PFS and OS **(Table 4).**

#### Multivariate analysis

**iPET4:** In the first multivariate analysis, interim PET metrics which were significantly associated with PFS and OS in univariate analysis were included.

Only rDS was found to be an independent predictor of PFS and OS. ΔSUVmax and ΔSUVmax + rDS were only independent prognostic factors on PFS but not for OS. DS and Menton 2011 criteria were not statistically significant at the multivariate level (**Table 5**). The second model of multivariate analysis included the three main interim PET metrics; rDS, DS, ΔSUVmax and clinical features which were significantly associated at univariate level. LDH rate was not included in the model because it is a component of IPI score. Menton 2011 criteria and ΔSUVmax + rDS were also not included because they were highly correlated with ΔSUVmax, and the latter is the only one to be validated. rDS, IPI score and chemotherapy were independent prognostic factors. DS and ΔSUVmax were not statistically significant at the multivariate level (**Table 5**).

**Table 5:** Multivariate analysis and log-rank test for PFS and OS at interim and end-of-treatment PET/CT.

	Multivariate analysis					
iPET4 (n = 181) 1st model	PFS			OS		
Variables	Cox regression analysis		Log rank test	Cox regression analysis		Log rank test
	HR	95%CI	*p-value*	HR	95%CI	*p-value*
**rDS**	14.23	[3.56–56.86]	0.0002	5.23	[2.96–9.25]	<0.0001
**ΔSUVmax**	3.33	[1.36–8.17]	0.008	NS	–	>0.1
**ΔSUVmax + rDS**	6.86	[1.37–34.13]	0.019	NS	–	>0.1
**Deauville score**	NS	–	>0.1	NS	–	>0.1
**Menton 2011 criteria**	NS	–	>0.1	NS	–	>0.1
**iPET4 (n = 181) 2nd model**						
**rDS**	4.46	[2.72–7.30]	<0.0001	2.71	[1.55–4.74]	0.0005
**IPI score**	2.54	[1.48–4.39]	0.0008	2.30	[1.19–4.46]	0.013
**Chemotherapy**	2.28	[1.16–4.49]	0.016	2.73	[1.19–6.24]	0.017
**ΔSUVmax**	NS	–	>0.1	NS	–	>0.1
**Deauville score**	NS	–	>0.1	NS	–	>0.1
**PET-eot (n = 165)**						
**rDS**	8.11	[4.68–14.05]	<0.0001	6.50	[3.49–12.12]	<0.0001
**IPI score**	2.01	[1.11–3.65]	0.02	2.21	[1.07–4.55]	0.03
**Deauville score**	NS	–	>0.1	NS	–	>0.1
**Chemotherapy**	NS	–	>0.1	NS	–	>0.1

PFS: Progression Free survival; OS: Overall Survival; NS: Not Significant; rDS: ratio Deauville Score = Tumour/Liver SUVmax ratio

Menton 2011 criteria: ΔSUVmax if eligible patient and DS if SUVmax initial tumour <10 and / or if SUVmax residual tumour >5

ΔSUVmax + rDS: ΔSUVmax if eligible patient and rDS if SUVmax initial tumour <10 and / or if SUVmax residual tumour >5

IPI: International Prognostic Index.

After 4 courses of chemotherapy, the combination of rDS and IPI shows a better prognosis in rDS-negative patients regardless of the initial IPIscore and a poor prognosis in rDS-positive patients regardless of the IPIscore.

**Fig 4:**
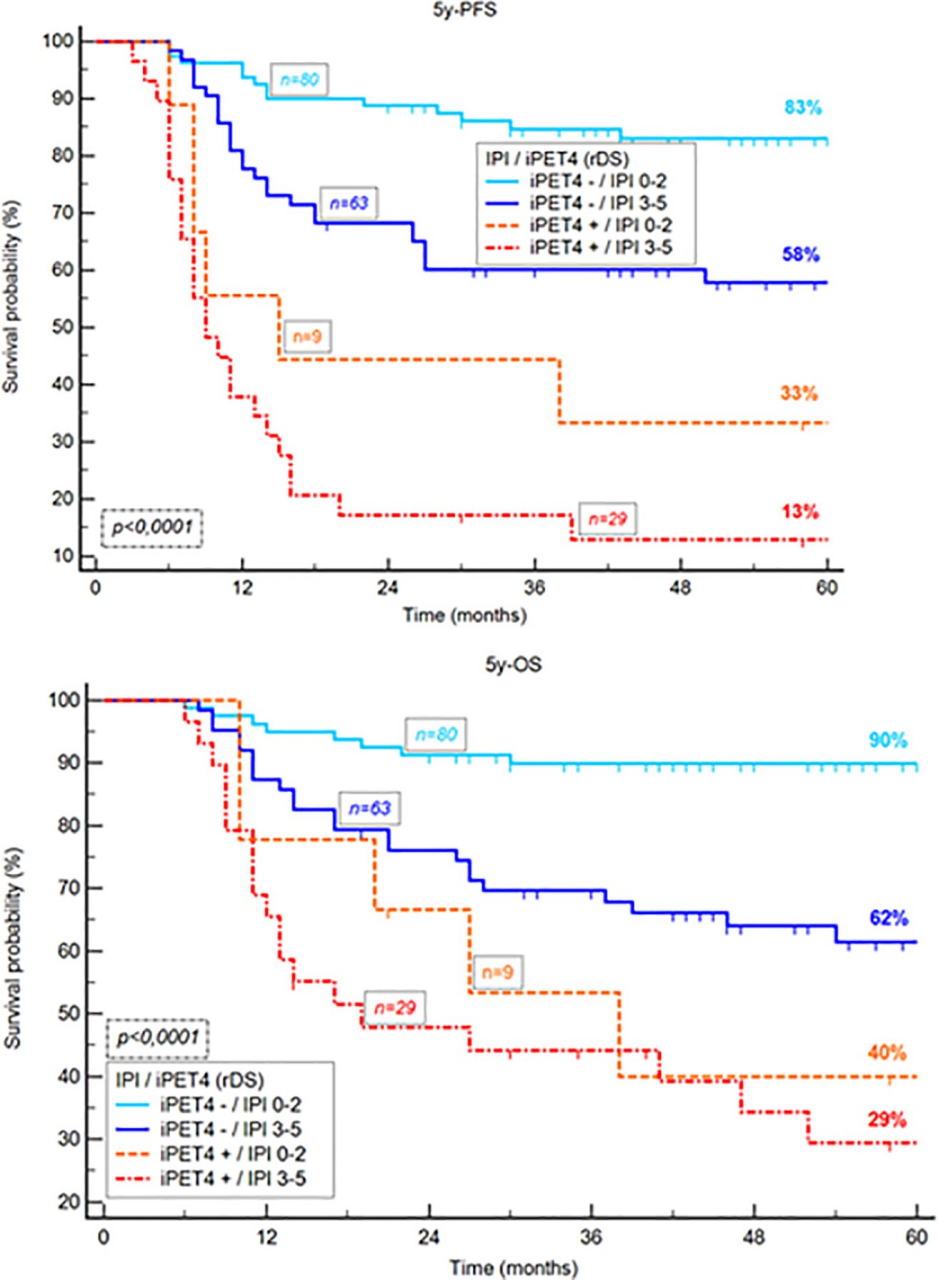
Kaplan-Meier estimates of progression-free and overall survival according to the rDS combined with IPI score.

However, combining rDS with IPI gave added predictive value. Four risk categories could be identified depending on the presence or absence of adverse factors: group 1 if rDS négative and IPI ≤2 (n = 80); group 2 defined as rDS negative and IPI >2 (n = 63), group 3 with rDS positive and IPI ≤2 (n = 9) and group 4 with rDS positive and IPI >2 (n = 29). These groups had significantly different outcome, with a respectively 5-year PFS of 83%, 58%, 33% and 13% (p < 0.0001) and a respectively 5-year OS of 90%,62%, 40% and 29% (p < 0.0001), (Fig 4).

**PET-eot:** Multivariate analysis revealed that rDS was the best independent prognostic factor for PFS and OS. IPI score was also statistically significant for all survival rates. DS and chemotherapy were not independent prognostic factors **(Table 5).**

## Discussion

The aim of our study was to evaluate the prognostic impact of the ratio between SUVmax of the hottest target residual lesion and SUVmax of the liver (rDS) in patients with DLBCL undergoing interim FDG-PET/CT (iPET4) during the first line of immunochemotherapy and PETeot and to compare rDS with criteria already validated *i*.*e* DS and ΔSUVmax on iPET4 and DS on PETeot. In our study, ROC analysis showed that the optimal cut-off for rDS was 1.4-fold of SUVmax liver on iPET4 and PET-eot. Prognosis accuracy using rDS has allowed a significant increase of PPV compared to Deauville criteria on iPET4 and PET-eot (81.58% vs. 67.79%, respectively, for PFS and 63.16% vs. 52.54%, respectively, for OS on iPET4; 94.12% vs. 75%, respectively, for PFS and 76.47% vs. 60.42%, respectively, for OS on PET-eot) and therefore decrease the number of false positives. The NPV of rDS remains satisfactory on iPET4 compared to DS (72.72% vs 75.41%, respectively, for PFS and 78.32% vs 80.32%, respectively, for OS) and comparable to DS on PET-eot (77.10% vs 77.77%, respectively, for PFS and 83.97% and 84.61%, respectively, for OS). This is the first study confirming the prognostic performance of Menton 2011 criteria, with an accuracy of 74.58% for PFS and 75.14% for OS. These results agree with the GAINED study, which shows the prognostic influence of iPET4 ΔSUVmax (using Menton 2011 criteria) in R-ACVBP and R-CHOP14 arms [23]. After four cycles of chemotherapy, rDS, Menton 2011 criteria and ΔSUVmax had the best specificity, PPV and accuracy better than DS. But only rDS was found to be an independent prognostic factor on PFS and OS. ΔSUVmax was statistically significant at the multivariate level on PFS but not on OS. Prognosis performances of ΔSUVmax + rDS (iPET4) were between the three mentioned above, and DS and showed no particular benefit. NPVs were similar for all evaluation criteria on PFS and OS on iPET4.

Only Zhang et al. have analysed the interest of tumor/liver ratio after four cycles and post-treatment PET/CT in DLBCL [17].

Their results showed that the optimal cut-off was 1.6-fold of SUVmax liver at PET4 and 1.4-fold of SUVmax liver at PET-eot. On iPET4, using a cut-off of 1.6, the PPV reaches 100% for PFS and 95% for OS and was far superior than our results (81.58% and 63.16%, respectively). The main hypothesis is that a higher cut-off increases specificity and then PPV. However, we improved statistical significance due to the doubling of the population (n = 79 for Zhang vs. 181 on iPET4 and n = 84 for Zhang vs. 165 on PET-eot). On PET-eot, using the same cut-off of 1.4, Zhang et al. reported a PPV of 100% for PFS and 50% for OS compared to 77% and 83.97%, respectively, in our study. Again, those differences could be explained by our larger population.

Our results suggest greater accuracy of outcome prediction with rDS. Kaplan-Meier survival prediction curves revealed that 5-year PFS and OS were 18% and 32%, respectively, in iPET4-positive patients vs. 72% and 77%, respectively, in iPET4-negative patients (p<0.0001). The 5-year PFS for patients with negative PET-eot was 76% vs. 6% for positive PET-eot and 5-year OS were 82% vs. 18% with p< 0.0001 for both PFS and OS, respectively. Interobserver agreement was perfect on iPET4 with ΔSUVmax (к = 1) and near perfect with rDS (к = 0.93) and DS (к = 0.85). On PET-eot к coefficient was better for rDS (к = 0.84) than DS (к = 0.56). These results agree with previous studies showing better interobserver agreement for ΔSUVmax (range: 0.74–0.92) than for DS (range: 0.53–0.8) [6, 7] and with Zhang et al. [17], who found greater reproducibility with use of SUVmax-liver-based method compared to DS (range 0.95 to 1 vs. 0.62 to 0.86, respectively). This can be explained by the more objective nature of measurements made for ΔSUVmax and rDS methods compared to visual interpretation of DS. Moreover, although a score 5 of DS has been defined as two to three times the liver SUVmax [3], no SUVmax cut-off has to be chosen to discriminate a score of 4 and 3. For this reason, only DS3 and 4 were considered to determine the optimal cut-off on ROC curves. Thus, a semiquantitative assessment of treatment response is likely to have a higher reliability and validity than visual analysis to predict outcomes.

Previously, two studies suggested a semiquantitative assessment with a tumor/liver ratio on iPET2. Itti et al. [19] found a cut-off between 1.25- to 1.4-fold of SUVmax-liver; more recently, Fan et al. [18] showed a cut-off of 1.6 leading to a more accurate reproducibility and outcome prediction. Similarly several authors proposed semiquantitative tumoral response in HL [15, 16] and in FL [20]. The tumor/liver ratio (rDS) has some important advantages: it is independent of the amount of administered activity and body weight, and it allows conversion of a visual qualitative scale (e.g., DS) to a continuous semiquantitative scale, and it allows the evaluation of iPET and PET-eot by binarizing responder and nonresponder patients by a defined semiquantitative cut-off point. rDS combines the benefits of DS and ΔSUVmax. Indeed, it can be applied without a baseline scan, regardless of a low baseline SUVmax (<10) or a high interim SUVmax (>5); overcomes the problem of reproducibility of a visual assessment; and allows a significant decrease of the number of false-positives. Our larger cohort confirms the hypothesis that an increase in background reference (liver) may improve the specificity, PPV and prognostic accuracy of iPET4 and PET-eot. However, the choice of appropriate evaluation criterion must be made according to the therapeutic decision. In the context of an escalation strategy, specific criterion with high PPV had to be chosen like rDS or ΔSUVmax. Inversely, a de-escalation strategy based on the results of PET should lead to use of a more sensitive criterion with better NPV, like DS.

Our data confirmed the predictive value of IPI for PFS and OS. A few studies have shown that combining clinical or biological factors with imaging (so-called integrative PET) allows us to identify different risk categories for patients with lymphoma [24, 25, 26]. In the present study, we have combined a clinical score, IPI and rDS, to stratify patients into four categories, according to the presence or absence of adverse factors. This has resulted in identifying four groups of patients with a significant different outcome. Especially, patients with a rDS positive iPET4 and IPI >2 had a very poor prognosis (5-year-PFS = 13% and 5-year-OS = 29%). With an equal IPI score, patients with a rDS positive iPET4 had a worse prognostic score.

We also confirmed that R-ACVBP immuno-chemotherapy improved survival compared to R-CHOP in young patients, as shown by Récher et al [27]. The benefit of this intensive chemotherapy found in this retrospective study may be related to this effect but also more likely to the fact that this regimen is only proposed to young patients, under 60 years of age.

This study’s main limitations are its retrospective and monocentric nature, and the heterogeneity of first-line treatment modalities could have affected the outcome. Although the liver SUVmean would theoretically be preferable over the liver SUVmax as a reference background, as this parameter is less noise-dependent, the liver SUVmax is the parameter currently used in clinical practice and in multicentre trials. Another technical problem could affect the computation of this ratio due to the improved performances of the new digital PET device. Technological advances in PET can lead to an important increase in not only the residual tumor SUVmax, but also the tumor/liver ratio [28].

## Conclusion

To conclude, rDS with a tumor/liver ratio of 1.4 is a robust prognostic factor in patients with DLBCL on iPET4 and PET-eot and shows better prognosis accuracy and survival prediction than Deauville criteria.

In addition, the combination of rDS with IPI score could be used for precise prediction of patient prognosis.

These findings may help guide the treatment and management of patients with DLBCL, although a prospective study is needed to confirm these data.

## Supporting information

S1 Dataset (XLSX)
